# Marine Carotenoids: Biological Functions and Commercial Applications

**DOI:** 10.3390/md9030319

**Published:** 2011-03-03

**Authors:** Carlos Vílchez, Eduardo Forján, María Cuaresma, Francisco Bédmar, Inés Garbayo, José M. Vega

**Affiliations:** 1 Algal Biotechnology Group, International Centre for Environmental Research (CIECEM), University of Huelva, 21760 Huelva, Spain; E-Mails: eduardo.forjan@dqcm.uhu.es (E.F.); maria.cuaresma@dqcm.uhu.es (M.C.); garbayo@uhu.es (I.G.); 2 Faculty of Business, University of Huelva, Plaza de la Merced 11, 21071 Huelva, Spain; E-Mail: f.bedmar@ono.com; 3 Plant Biochemistry and Molecular Biology Department, Faculty of Chemistry, University of Seville, 41012 Seville, Spain; E-Mail: jmvega@us.es

**Keywords:** carotenoids, microalgae, applications, nutraceuticals, health benefits

## Abstract

Carotenoids are the most common pigments in nature and are synthesized by all photosynthetic organisms and fungi. Carotenoids are considered key molecules for life. Light capture, photosynthesis photoprotection, excess light dissipation and quenching of singlet oxygen are among key biological functions of carotenoids relevant for life on earth. Biological properties of carotenoids allow for a wide range of commercial applications. Indeed, recent interest in the carotenoids has been mainly for their nutraceutical properties. A large number of scientific studies have confirmed the benefits of carotenoids to health and their use for this purpose is growing rapidly. In addition, carotenoids have traditionally been used in food and animal feed for their color properties. Carotenoids are also known to improve consumer perception of quality; an example is the addition of carotenoids to fish feed to impart color to farmed salmon.

## 1. Marine Carotenoids: Biological Functions and Benefits to Human Health

In photosynthetic organisms including plants and microalgae, carotenoids play various roles. Essentially, carotenoids may act as accessory pigments in light harvesting functions during the light phase of photosynthesis and are also able to photoprotect the photosynthetic machinery from excess light by scavenging reactive oxygen species (ROS) like singlet oxygen and other free radicals [1].

In humans, the most relevant biological functions of carotenoids are linked to their antioxidant properties, which directly emerge from their molecular structure. In recent years, the understanding of ROS-induced oxidative stress mechanisms and the search for suitable strategies to fight oxidative stress has become one the major goals of medical research efforts. A number of studies have been reported which implicate oxidative stress involvement in degenerative pathogenesis, e.g., Alzheimer and Parkinson [1,2]. In parallel, a carotenoid-enriched diet has been found to diminish the risk of suffering from degenerative diseases [2].

Moreover, far from being just a speculative hypothesis, the benefits of carotenoids (lutein, β-carotene, lycopene) to human health have been shown based on the positive impacts of the antioxidant bioactivity of carotenoids in inmunoresponse modulation, in signaling transduction between cells and in anti-inflammatory response mechanisms [3–5]. These positive consequences are the result of either the direct chemical action of carotenoids on biological molecules and structures or through expression of different genes involved in antioxidant responses [2]. The main biological functions of carotenoids and benefits to health are listed in Table 1.

### 1.1. Provitamin A Activity

One of the most important functions of carotenoids in the human body is their ability to convert into retinol (provitamin A function), a faculty that about 10% of carotenoids identified in nature possess [6]. Vitamin A is well recognized as a factor of great importance for child health and survival, its deficiency causes disturbances in vision and various related lung, trachea and oral cavity pathologies [7]. Animals and humans cannot synthesize carotenoids *de novo*, although they are able to convert them into vitamin A. Diet is the only source for these precursors for retinol synthesis, fruits, vegetables and microalgae being the major suppliers of provitamin A active carotenoids. As a reference value, a recommended daily intake of 6 mg of carotenoids has been proposed. This value is based on the contribution of compounds with provitamin A activity, specially β-carotene, which has been assigned a provitamin A activity of 100% [8].

### 1.2. Carotenoids and Cancer

In recent years, epidemiological evidence supporting a protective effect of carotenoids to the development of chronic and degenerative diseases has grown considerably. We must not forget that cancer and cardiovascular diseases are the leading causes of death in the world and that approximately 50% of all tumors are attributed to the diet [9].

From a nutritional point of view, an antioxidant can be defined as any substance present in foods that significantly reduces the adverse effects of reactive oxygen species in normal physiological conditions in humans [10]. Antioxidants, in particular carotenoids, are essential for cell health due to their protective action on cellular components against oxidative damage [2]. These activities have generated two lines of research related to the physiological functionality of carotenoids: on one hand, their activity as membrane antioxidants, therefore involved in the oxidative cell cycle [11] and, on the other hand, their involvement in control processes of cell differentiation and proliferation [12]. As an example of the first, a recent study showed that antioxidant enzymes including catalase, superoxide dismutase and peroxidase levels in plasma and liver of mice increased significantly when the animals were fed with microalgae biomass (*Haematococcus pluvialis*, *Scenedesmus platensis* or *Botryococcus braunii*), which reveals an increased antioxidant protection against free radicals [13].

It is well known that cellular proliferation is controlled by the communication established between the cells in a tissue. Cell communications reset or stimulation becomes essential if abnormal cell proliferation occurs. In that respect, it has been mentioned that carotenoids might stimulate expression of genes directly involved in regulation of cell communication processes. In more detail, carotenoids would directly act on DNA in order to regulate the production of RNA that is responsible for *gap-junctions* communications, which could successfully explain some anti-tumor activities of carotenoids [2,12]. Immune system cells also require intercellular communication to conduct their activity efficiently, so the previous action mechanism of carotenoids could also apply for supporting the immune system activity. As an example, high doses of β-carotene increase the CD4 to CD8 lymphocyte ratio, which is very low in patients suffering from HIV disease [14].

In the last decades, many laboratory and epidemiological studies have been conducted which suggest that intake of carotenoids and cancer prevalence are inversely related [4,15–17]. Among the carotenoids, lycopene has been one of the most extensively studied [4,18–20] probably due to the greater anticancer capacity shown with respect to other carotenoids [21]. Within the wide frame of research carried out by Giovannucci *et al.* [4], lycopene intake and prostate cancer were found to be inversely related. The inverse relation was based on *in vivo* and *in vitro* studies on the effect of lycopene in tumor cell lines that showed tumor cells growth inhibition by the action of lycopene [19,20]. Although the functional meaning of the lycopene distribution in the organism has not been fully elucidated, it is particularly interesting that this carotenoid predominates in testes and adrenal glands, with an abundance of about 60 to 80% of the total carotenoids [22]. It has also been inferred that astaxanthin could be effective against benign prostatic hyperplasia and against prostatic cancer through inhibition of the enzyme 5-a-reductase which is involved in abnormal prostate growth [2,23].

Antitumoral activity of carotenoids toward other type of cancer has also been observed. In particular, β-carotene, astaxanthin, cantaxanthin and zeaxanthin have been shown to promote reduction in size and number of liver neoplasias *in vivo* [21,24]. Other studies have shown that inclusion of carotenoids in the diet and reduced risk of colon cancer might be directly related [25–27]. The antitumoral effect of b-carotene has also been associated to the nutritional situation of the studied population. As an example, β-carotene implementation studies carried out at Linxian (China) in population that suffered from a diet deficient in vitamins and mineral salts, led to reduced incidence of total mortality from gastrointestinal cancer [28]. Interestingly, in population not affected by nutritional deficiency but included in cancer risk groups (e.g., smokers or asbestos-exposed groups) it has been shown that β-carotene supplements even might increase cancer risk, probably due to generation of metabolites that increase the cell oxidative state and led to reduced control of cell differentiation and cell proliferation processes [29–32].

Although carotenoids including zeaxanthin, criptoxanthin and lutein antitumoral activities have still been scarcely studied, the strategy of using carotenoids as chemoprotecting agents is not yet endorsed by clinical trials. More on the contrary, in spite of using β-carotene as pure drug for producing an intense punctual effect after any dosage intake, the derived positive action of carotenoids should be produced through continuous intake of usual quantities. This idea is in line with current dietary recommendations that suggest consumption of five fruit and vegetables portions a day, which will provide water, vitamins, fiber and phytochemical compounds including carotenoids in sufficient quantities to meet our body needs [10,16].

### 1.3. Carotenoids and Cardiovascular Diseases

Cardiovascular diseases are the leading cause of death in developed countries, and have become the main health problem also in developing countries [33]. These include acute myocardial infarction and disorder of high morbidity and mortality [34]. Oxidative stress and inflammation are the main factors contributing to the pathophysiology of these disorders [35,36]. In particular, the oxidative stress induced by ROS can cause low density lipoproteins oxidation (LDL), an aspect that plays a key role in the pathogenesis of atherosclerosis [37,38].

Another major feature of carotenoids is protection of LDL against oxidation [39,40], which confers carotenoids antiatherogenic properties [2,36,41]. In addition, carotenoids have been shown to inhibit *in vivo* lipid peroxidation processes [42], by which the presence of carotenoids in cell membranes is essential to act as stabilizing elements of these structures [8,43]. In this sense, the antioxidant activity of some carotenoids during radical peroxide-induced cholesterol oxidation was investigated by Palozza *et al.* [44], showing that carotenoids exerted a significant antioxidant activity, in the decreasing activity order indicated: astaxanthin, cantaxanthin, lutein and β–carotene. Several authors have published that daily dietary β-carotene supplementation in mammals led to decreased plasma levels of total lipids, cholesterol and triglycerides [45,46].

Numerous epidemiological studies suggest that diets rich in carotenoids could protect the human body from certain cardiovascular diseases due to the involvement of oxidizing substances and oxidative stress in the development and clinical expression of coronary heart disease [47]. In fact, high lycopene levels in plasma and tissues have been inversely linked to coronary heart disease [48], myocardial infarction [49] and risk to suffer from arteriosclerosis [50]. Low lutein levels in plasma have also been associated with an increased tendency to suffer from myocardial infarction [51], while a high intake of lutein has been inversely related with the risk of stroke [52].

Likewise, low α-carotene levels in serum have been shown to inversely correlate prevalence of coronary artery disease and formation of arterial plaque, by which α-carotene has been proposed as a potential marker for human atherosclerosis. In addition, carotenoids displaying high levels of provitamin A activity, including α-carotene, β-carotene and β-cryptoxanthin, have been associated with reduced risk of angina pectoris disease [53,54]. Other epidemiological studies have also found low levels of oxygenated carotenoids (namely xanthophylls: lutein, zeaxanthin, lycopene, β-cryptoxanthin, β-carotene and α-carotene) in plasma of patients with acute and chronic coronary syndromes [55,56]. Particularly, in the recent study by [38], high levels of β-cryptoxanthin and lutein in plasma have been shown to decrease risk for suffering from myocardial infarction, but no statistically significant associations with other carotenoids were found.

### 1.4. Carotenoids and Eye Health

Many research studies showed that lutein and zeaxanthin are the main responsible pigments for both the yellowing and the maintenance of normal visual function of the human eye macula [57,58], while other major carotenoids in serum (α-carotene, β-carotene, lycopene and β-cryptoxanthin), are absent or are found in trace amounts in the human macula [59]. In the eye macula, lutein and zeaxanthin absorb blue light and also attenuate pernicious photooxidative effects caused by the excess blue light, while reducing eye chromatic aberration. Due to their antioxidant properties, carotenoids protect the eye macula from adverse photochemical reactions [60]. In people over the age of 64, visual sensitivity directly depends on lutein and zeaxanthin concentrations in retina [61].

Major prevalency of cataracts has also been linked to people with low levels of lutein and zeaxanthin [62]. Also macular degeneration, the main cause of irreversible loss of vision in people above 65 years in industrialized countries, has been associated with very low levels of lutein and zeaxanthin [63,64].

The spectra of lutein and zeaxanthin show a wide absorption band with a peak at 450 nm, which is thought to be involved in absorbing excess blue light before it comes to photoreceptors, therefore preventing the eye macula from being damaged by blue light [65]. Moreover, due to lutein’s and zeaxanthin’s biophysical and biochemical properties for ROS scavenging, these carotenoids might also preserve the membrane structure in the eye photoreceptors from lipid peroxidation processes [66], in contrast to non-polar carotenoids as lycopene and β-carotene [67]. Concentration of lutein and zeaxanthin in the retina can be increased on diet bases (spinach and maize) and on supplements of both pigments [60,68].

### 1.5. Other Physiological Functions of Carotenoids

Carotenoids provide skin photoprotection against UV light [69–71]. Due to their scavenging action on ROS, carotenoids also possess anti-inflammatory properties [72–74]. In this sense, it has been recently described that astaxanthin raises anti-inflammatory effects while preserving essential lipids and proteins of human lymphocytes [74]. Astaxanthin would act by inducing superoxide dismutase and catalase enzyme activities [74]. Other studies have shown astaxanthin to protect from CCl_4_-induced hepatic damage by inhibiting lipid peroxidation, stimulating the cellular antioxidant system and modulating the inflammatory process [73]. Table 1 resumes biological functions, benefits to health and applications of the main carotenoids, including their role in prevention of cataracts [75,76], macular degeneration [77–80], retinitis [81–83] and gastric infection [84].

Carotenoids have been used as preservatives in cosmetics and, combined with other antioxidants or algal bioactive substances, also in creams and lotions for sun protection [85]. The beneficial effect of carotenoids has also been shown in patients with psoriasis, skin inflammatory pathology. Lima and Kimball [86] found low levels of carotenoids in the skin correlate well with psoriasis prevalence.

Finally, it is interesting to note that, in recent years, carotenoids are being considered as important protective molecules in gastric disorders. It has been published that a high intake of carotenoids prevents the development of disorders caused by *Helicobacter pylori* [84,87,88], a Gram negative bacteria genus that colonizes the gastric mucosa of at least half of the human population [89].

## 2. Marine Carotenoids: Applications

Carotenoids have been traditionally used in food and animal feed due to their color properties. The natural carotenoids are used to reinforce fish color, which increases consumers’ perception of quality. An example is the addition of carotenoids to fish feed to impart color to farmed salmon. The nutraceutical properties of carotenoids also attracted attention of the food industry. Large numbers of scientific studies have confirmed the benefits of carotenoids to health and use for this purpose is growing rapidly. Besides, carotenoids have been proposed as added-value compounds that could contribute to make microalgal biofuel production economically feasible [90,91].

Among all existing natural carotenoids, five can be considered to be the most relevant ones in economical terms (Table 2). The main applications of carotenoids are currently as dietary supplements, fortified foods, food color, animal feed and pharmaceuticals and cosmetics.

β-carotene, the most widely known of the carotenoids, is known to be a vitamin A precursor, likely several other carotenoids. Carotenoids have antioxidant properties and a large number of studies have confirmed their benefits to health. In particular, carotenoids are thought to reduce the risk of degenerative diseases and cancer especially in elderly people, as explained above [29,32,41].

The health industry uses carotenoids in over-the-counter (OTC) dietary supplements and fortified foods. This is one of the fastest growing segments of the industry but is still relatively small compared to the color segment. The pharmaceutical and cosmetics industries also use carotenoids mainly for their coloring properties, though their use by the pharmaceutical and cosmetics companies is growing rapidly due to their nutraceutical properties. An example of a new product from this segment is a ‘beauty pill’ containing the carotenoid lycopene. This product belongs to a new market segment known as ‘cosmeceuticals’, which aims to combine cosmetics and nutraceutical food ingredients to create products to improve skin and hair.

Chemically synthesized nature identical carotenoids dominate the market but naturally extracted carotenoids are growing in popularity due to increasing demand for natural products from consumers. Natural carotenoids can be extracted from plant material such as tomatoes, algae and fungi. Individual carotenoids are available in a variety of forms. The most common forms are cold water soluble powder, oil emulsion and beadlets. Concentrations range from 0.2 to 100%. The most common concentration is 10%. Blends or mixed carotenoids are also available containing two or more different carotenoids. Like the individual carotenoids, blends are available in a variety of forms including, water dispersible powder, oil suspension and beadlet forms. The concentration of blends ranges from 1 to 30%, with the most common concentration being 10% [91–93].

### 2.1. Dietary Supplements and Food Color

Carotenoids are widely used as color enhancers in natural foods including egg yolk, chicken meat or fish [90]. However, among more than 400 known carotenoids just few of them have been commercially used, including β-carotene, lycopene, asthaxanthin and lutein [91]. One of the main advantages in the use of microalgae as a carotenoid carrier in the food industry is that many other antioxidant compounds present in the microalgal biomass have positive impact on human health, sometimes acting with carotenoids synergistically [92].

In addition, if carotenoids are disposed within the microalgal matrix (carotenoid enriched dry biomass) also a number of minerals whose presence is inherent to the algal biomass are provided in the formula. These mineral have positive effects to human health, especially in enhancing anabolic activities. Carotenoids have also been used as preservatives in cosmetics and solar protection products [85].

Because of the content of carotenoids, the commercial value of microalgae increased and their use extended widely into many applications of the food market. That includes the use of *Arthrospira*, *Chlorella*, *Dunaliella, Spirulina* and *Aphanizomenon* as functional foods which can be found in the market in the form of pills, tablets and capsules. These microalgae have also been integrated in nutritional formula of pasta, snacks, sweets, drinks and bubble gum [91,93].

Microalgae are also used in fish color quality improvement in aquaculture. Salmonids are supplied with astaxanthin-enriched microalgae species, in particular *Haematococcus pluvialis* [2].

### 2.2. Environmental Applications: Carotenoids in Biorefining

Microalgae have gained interest as promising feedstocks for biofuels. The productivity of these photosynthetic microorganisms in converting carbon dioxide into carbon-rich lipids greatly exceeds that of agricultural oleaginous crops, without competing for arable land. However, large scale production of lipid-enriched algal biomass is not yet economically feasible and still requires major efforts in developing suitable technology which allows for reducing biomass production costs at large scale by at least an order of magnitude. Recent advances in systems biology, genetic engineering and methods to profit from the fractions of the biomass residue open new scenarios to make biofuel production from microalgae economically suitable within a period of about 15 years. Production of biodiesel and other bio-products from microalgae can be more cost-effective and profitable if combined with processes such as wastewater and flue gas treatments [94,95]. Carotenoids are, indeed, one of the main bio-products whose production is required to make biofuel production economically feasible. The paradox, therefore, is that production of high-added value compounds as carotenoids should so far be the only way to approach economical production of a low value energy source as biofuel from microalgae.

### 2.3. Commercial Value for Carotenoids

In recent years, production of carotenoids has become one of the most successful activities in microalgal biotechnology. The demand for carotenoids obtained from natural sources is increasing. This has promoted major efforts to improve carotenoid production from biological sources instead of chemical synthesis [96]. According to the report published by Business Communications in March, 2008, the global market for all commercial carotenoids accounted for 766 million dollars, with expectations of rising to 919 million dollars in 2015. In particular, beta-carotene market volume in 2007 was 247 million dollars, with expectations of reaching 285 million dollars in 2015. Besides lycopene and β-carotene, xanthophylls lutein, astaxanthin and cantaxanthin appear as the most demanded and valuable carotenoids. Astaxanthin market volume in aquaculture in 2009 was 260 million dollars and about 2500 $ kg^−1^. In addition, lutein market volume in 2010 accounted for about 190 million dollars, the carotenoid experiencing the most rapid growth in sales [97]. Therefore, carotenoid-containing microalgae find many applications in a wide range of commercial activities, the reason for which carotenoid-enriched microalgae production is steeply becoming an attractive business (Table 3).

## Figures and Tables

**Table 1.** Biological functions, benefits to health and applications of the main carotenoids.

**Carotenoid**	**Functions and benefits to health**	**References**
*Lycopene*	In prostatic hyperplasia and prostate cancer	[19–21]
	In the prevention of atherosclerosis and acute and chronic coronary syndromes	[48–50,55,56]
*β–carotene*	Provitamin A function	[6,8]
	In colorectal cancer	[25–27]
	In the prevention of acute and chronic coronary syndromes	[53–56]
	Photoprotection of skin against UV light	[69–71]
*Astaxanthin*	In benign prostatic hyperplasia and prostate and liver tumors	[2,21–24]
	Anti–inflammatory properties	[72–74]
*Zeaxanthin*	Active against liver neoplasms	[21,24]
	In the prevention of acute and chronic coronary syndromes	[55,56]
	Helps to maintain a normal visual function	[57,58]
	In the prevention of cataracts	[62,75,76]
	To prevent macular degeneration associated with age	[65,77–80]
*Lutein*	In the prevention of acute and chronic coronary syndromes and stroke	[38,55,56,66]
	Helps to maintain a normal visual function	[57,58]
	In the prevention of cataracts	[62,75,76]
	To prevent macular degeneration associated with age	[65,77–80]
	In the prevention of retinitis	[58,81–83]
	To avoid gastric infection by *H. Pylori*	[84]

**Table 2.** Main commercial carotenoids and origin.

**Carotenoid**	**Origin**
β-carotene	Synthetic and naturally extracted forms [6,22].
Astaxanthin	Synthetic nature identical and naturally extracted forms [2,13]
Canthaxanthin	Comes in synthetic nature identical form [13].
Lycopen	Only currently available in natural form [22]
Lutein	Only comes in natural form [6,13,85].

**Table 3.** Main applications of microalgae due to their carotenoid content.

**Microalga**	**Application**	**Product formula**	**Price**
*Chorella vulgaris*	Aquaculture, cosmetics, nutraceuticals, food ingredient	Dry powder, tablets	$30–100 kg^−1^
*Isochrysis galbana*	Aquaculture, cosmetics, nutraceutical	Paste, dry powder	$100–400 kg^−1^
*Nannochloropsis gaditana*	Aquaculture, cosmetics	Paste, dry powder	$300 kg^−1^
*Pavlova lutheri*	Aquaculture	Paste, dry powder	>$300 kg^−1^
*Phaeodactylum tricornutum*	Aquaculture, nutraceuticals	Paste, dry powder	>$200 kg^−1^
*Tetraselmis*	Aquaculture	Paste, dry powder	$600–800 kg^−1^
*Thalassiosira weissflogii*	Aquaculture	Paste, dry powder	>$300 kg^−1^
*Arthrospira*	Cosmetics, nutraceuticals	Paste, dry powder	>$200 kg^−1^
*Haematococcus pluvialis*	Aquaculture, nutraceuticals	Dry powder	>$600 kg^−1^
*Dunaliella salina*	Nutraceuticals, food ingredients	Dry powder, tablets	$100–400 kg^−1^
